# P04.61. Do infectious disease (ID) physicians use cranberry for prevention of urinary tract infections (UTI)?

**DOI:** 10.1186/1472-6882-12-S1-P331

**Published:** 2012-06-12

**Authors:** K Shere-Wolfe, J Tilburt, C D'Adamo, B Berman, M Chesney

**Affiliations:** 1 University of Maryland, Baltimore, USA; 2 Mayo Clinic, Rochester, USA; 3 University of California, San Franscisco, San Francisco, USA

## Purpose

The purpose of this study was to survey how ID physicians view cranberry for UTI prevention.

## Methods

In a 2010 survey of 1000 ID physicians, we presented a case vignette about prevention of uncomplicated UTIs. “Ms. Smith is a 23 y.o. female who presents with a history of recurrent bacterial UTIs. In the past, she has self-treated with antibiotics at the first sign of infection; however she is now interested in preventing future occurrences. Previous work up has revealed no predisposing factors.” We also presented a research summary of an article demonstrating evidence of efficacy of cranberry extract for UTI prevention.

## Results

The overall response rate for the survey was 31%. There were 292 responses for the question “Which ONE option best reflects your usual choice of initial management of UTIs in Ms Smith?”. Fifty-four percent of respondents chose antimicrobial prophylaxis, 29% chose cranberry, and 17% chose “other”. There were 298 responses for the question “Based on the research summary, how would you rate the comparative effectiveness of cranberry product versus trimethoprim for Ms Smith?”. The majority of respondents found cranberry equally (82%) or more (4%) beneficial. Five percent felt that cranberry was less beneficial and 9% felt that it was not at all beneficial. Finally, 301 participants responded to the question “Based on the information given, which ONE statement best characterizes your impressions about integrating cranberry products into the treatment plan for Ms. Smith?”. Almost half (49%) of respondents would consider using cranberry if the patient expressed an interest but only 22% would prescribe cranberry routinely. Twenty-five percent were not sure if they would use cranberry and 4% would not use cranberry.

## Conclusion

Despite acknowledgement of the comparative efficacy of cranberry versus antimicrobial prophylaxis, the majority of ID physicians are not willing to prescribe cranberry routinely.

